# Overestimating Overdiagnosis in Breast Cancer Screening

**DOI:** 10.7759/cureus.966

**Published:** 2017-01-09

**Authors:** Alan Hollingsworth

**Affiliations:** 1 Mercy Breast Center, Mercy Hospital - OKC

**Keywords:** overdiagnosis, invasive breast cancer, epidemiology, autopsy prevalence studies, length bias, mammographic screening

## Abstract

Overdiagnosis in breast cancer has been a focus of increasing concern with wide ranges of calculations made indirectly through the study of prospective randomized trials and analyses of large registries. While most admit that some degree of overdiagnosis is inherent with ductal carcinoma in situ (DCIS), the rate of overdiagnosis with invasive disease is highly controversial. Although it is generally accepted that overdiagnosis is calculated through indirect means and deductive reasoning, this is not entirely the case. Patients who refuse treatment, yet curiously return for follow-up, allow a direct glimpse at the natural history of screen-detected cancers. And historic autopsy studies offer information as to undiagnosed disease prevalence from the pre-screening era. While these autopsy studies support a modest degree of overdiagnosis in DCIS, they do not support widespread overdiagnosis for invasive cancer. The 1.3% mean incidence of invasive disease from seven autopsy studies correlates closely with disease prevalence, a direct observation that cancers do not remain quiescent in the breast until death. If invasive breast cancer does not regress in untreated patients and does not remain quiescent, then the high estimates being calculated for overdiagnosis are more likely to be length bias from long natural histories rather than true overdiagnosis.

## Editorial

Overdiagnosis of invasive breast cancer through mammographic screening is based on indirect evidence that draws its final conclusion through deductive reasoning. Attempts to counter these conclusions are met with seemingly plausible answers. For instance, one of the most common criticisms against the high calculated rates of overdiagnosis is the failure to correct for lead time at the tail end of screening trials. If lead time correction is accomplished, then overdiagnosis of invasive breast cancer almost disappears. Yet, the epidemiologists who actively promote overdiagnosis as fact are quick to point out that the very notion of lead time assumes that all invasive breast cancers progress over time, which may not be the case (forming a perfect circle of reason). To bolster that claim, more indirect evidence is generated to “prove” that many invasive cancers do not progress.

In a recent article in the New England Journal of Medicine [1], H. Gilbert Welch and associates draw from the Surveillance, Epidemiology, and End Results (SEER) program, spanning the years from 1975 to 2012, where they estimate the extent of overdiagnosis in mammographic screening. Using yet another variation of indirect reasoning, this time focusing on tumor size, it was determined that “only 30 of the 162 additional small tumors per 100,000 women that were diagnosed were expected to progress to become large,” implying that as many as 81.5% of women could be overdiagnosed by screening. This conclusion sets a record high for the calculated extent of overdiagnosis. Given that there are no patient-specific data about mammography use and compliance in these massive SEER reviews, one has to wonder how far these indirect methodologies can take us. Are we headed to 100% overdiagnosis?

One question always looms in this debate when addressing the overdiagnosis of invasive breast cancer (as distinct from DCIS)—why don’t we see evidence of these overdiagnosed tumors clinically? Of course, the epidemiologists who work to inflate overdiagnosis have beaten that argument down to their own satisfaction by reminding us that these overdiagnosed tumors are all removed, making measurement impossible.

Still, what is happening at the level of the individual patient? At the level of histology and tumor biology? For such an allegedly pervasive phenomenon, there must be a clinical correlate. In fact, the only true overdiagnosis that can be directly documented is more accurately labeled as misdiagnosis, e.g., when a complex sclerosing lesion is confused with invasive carcinoma, something that can trip up even the experts [2]. But this scenario doesn’t even begin to explain the staggering numbers for overdiagnosis being generated indirectly and, as a result, spilling into proposed informed consents for screening.

Using the strict definition of overdiagnosis—tumors that never progress—there are only two scenarios that could be happening at the histologic level, that is, tumor regression or, alternatively, tumor quiescence where the cancer simply reaches a certain size and stops. Either way, the tumor would not become clinically evident during the life of the patient.

Of these two possible explanations, the focus recently has been more on complete tumor regression, a shifty target since the evidence has already disappeared. Dr. Welch and associates will sometimes reference themselves in claiming that evidence supports regression [3], yet these are not studies where direct observation confirms that cancers melt away. Instead, the authors simply layer more indirect evidence on top of the indirect premise, creating a circle of self-validation.

A different perspective emerges through direct patient care. While there is some evidence histologically for “burned out” DCIS, the microscopic evidence from which to theorize the same for invasive regression is scant, at best. In contrast to the claim that we can only use indirect methods to study overdiagnosis, we clinicians have the occasional opportunity to witness potential tumor regression in the clinic by virtue of those women who refuse biopsy of screen-detected abnormalities. Or, after biopsy, patients refuse further treatment. Curiously, some will return for follow-up a year or more later, often still declining further intervention. For us, this sequence happens once or twice a year, mounting to a fair number of instances over an extended time period. The collective experience of thousands of radiologists over decades seems to be similar to our facility where we have never seen a single case of invasive tumor regression in the untreated patient.

The more plausible explanation would be the option of tumor quiescence—that is, indolent tumors that reach a certain size and stop growing, remaining silent for the life of the patient. The easily accessible repository in which to document this phenomenon—directly—would be autopsy studies, in which these cancers should be evident in numbers out of proportion to expected disease prevalence. Oddly, there is so much momentum by the overdiagnosis bandwagon that it is not uncommon to hear stated that autopsy studies of occult breast cancer show “pretty much the same” as what one sees in prostate cancer where overdiagnosis is readily evident. This is not the case.

In the 1990s, there was enormous enthusiasm for the detection and treatment of DCIS, as it was felt that we could nip invasion in the bud every time. To counter this trend, an epidemiologist set about to show how common DCIS could be found in autopsy studies. Indeed, the review [4] indicated a modest potential for overdiagnosis in DCIS (range 0–14.7% in seven autopsy studies, drawn largely from the pre-mammographic era). As one would expect, the higher rates were seen when more slides were examined, the mean number of slides per breast in the studies ranging from nine to 275.

Seemingly forgotten today, this comprehensive autopsy review secondarily included coverage of invasive disease as well. Given that undiagnosed disease prevalence in the United States for invasive breast cancer among the living is around one percent in an unscreened population (based on three-fold incidence), one would expect the autopsy data to generate a number well in excess of one percent if widespread tumor quiescence were at work. In fact, the discovery of occult invasive breast cancer in these seven autopsy studies ranged from 0 to 1.8%, with a median of 1.3%, a strong indictment against the alleged phenomenon of tumor quiescence. Of special interest, the lead author of this 1997 autopsy review was H. Gilbert Welch, cutting his teeth on DCIS, who many years later would move on to invasive disease. In 2012, Bleyer and Welch would claim that, over the past 30 years, 1.3 million women in the United States have been overtreated for pseudocancers of the breast and that we continue at the rate of 70,000 per year [5].

If neither tumor regression nor tumor quiescence is at work, then most likely, the magnified illusion of overdiagnosis is occurring due to old-fashioned length bias with very long natural histories for many breast cancers. By using indirect methodology, these tumors appear as “excess cancers” when adjustments are not made accordingly.

Overdiagnosis is intertwined with length bias—the former being tumors that never progress, the latter relating to tumors that slowly progress. Yet, the two cousin concepts can be indistinguishable in large-scale population studies, depending on how the data is handled. And given the choice of picking overdiagnosis as one’s operative word versus length bias, which is the more powerful iconoclastic bombshell? “Overdiagnosis” sends shivers up the public spine, whereas “Length Bias” only draws a collective yawn.
